# Fatal Methanol Poisoning Caused by Drinking Adulterated Locally Distilled Alcohol: Wakiso District, Uganda, June 2017

**DOI:** 10.1155/2020/5816162

**Published:** 2020-04-28

**Authors:** Birungi Doreen, Patricia Eyu, Denis Okethwangu, Claire Biribawa, Susan Kizito, Miriam Nakanwagi, Joyce Nguna, Innocent H. Nkonwa, Denis N. Opio, Freda L. Aceng, Phoebe H. Alitubeera, Daniel Kadobera, Benon Kwesiga, Lilian Bulage, Alex R. Ario, Bao-Ping Zhu

**Affiliations:** ^1^Uganda Public Health Fellowship Program, Kampala, Uganda; ^2^US Centers for Diseases Control and Prevention, Kampala, Uganda; ^3^Division of Global Health Protection, Center for Global Health, US Centers for Disease Control and Prevention, Atlanta, GA, USA

## Abstract

**Background:**

Methanol, an industrial solvent, can cause illness and death if ingested. In June 2017, the Uganda Ministry of Health was notified of a cluster of deaths which occurred after drinking alcohol. We investigated to determine the cause of outbreak, identify risk factors, and recommend evidence-based control measures.

**Methods:**

We defined a probable case as acute loss of eyesight and ≥1 of the following symptoms: profuse sweating, vomiting, dizziness, or loss of consciousness in a resident of either Nabweru or Nangabo Subcounty from 1 to 30 June 2017. In a case-control study, we compared exposures of case-patients and controls selected among asymptomatic neighbors who drank alcohol and matched by age and sex. We collected alcohol samples from implicated bars and wholesaler *X* for testing.

**Results:**

We identified 15 cases; 12 (80%) died. Among case-patients, 12 (80%) were men; the median age was 43 (range: 23–66) years. Thirteen (87%) of 15 case-patients and 15 (25%) of 60 controls last drank a locally distilled alcohol at one of the three bars supplied by wholesaler *X* (OR_M-H_ = 15; 95% CI: 2.3–106). We found that alcohol sellers sometimes added methanol to drinking alcohol to increase their profit margin. Among the 10 alcohol samples from wholesaler *X*, the mean methanol content (1200 mg/L, range: 77–2711 mg/L) was 24 times higher than the safe level.

**Conclusion:**

This outbreak was caused by drinking a locally distilled alcohol adulterated with methanol from wholesaler *X*. We recommended enforcing existing laws governing alcohol manufacture and sale. We recommended timely intravenous administration of ethanol to methanol poisoning victims.

## 1. Background

Acute methanol poisoning leads to serious health consequences in humans. Sporadic methanol poisonings occur either intentionally through the abuse of methanol-containing fluids or unintentionally through the misuse of products containing methanol as a solvent. Occasionally, methanol poisoning occurs when liquor is adulterated with methanol. Once ingested, methanol is metabolized by hepatic alcohol dehydrogenase enzyme (ADH) to the highly toxic formic acid, which inhibits mitochondrial respiration [1]. The accumulation of formic acid leads to metabolic acidosis causing damage to the optic nerve and retinal nerve fibers, leading to loss of vision, and necrosis of basal ganglia, leading to irreversible neurologic damage and death [2]. Outbreaks of methanol poisoning have been reported worldwide, from Asia to Europe to Africa. The sizes of these outbreaks have ranged from 20 to > 1000 victims, with case-fatality rates of > 30% in some instances [3].

On 23 June 2017, the Uganda Ministry of Health (MoH) through the Public Health Emergency Operations Centre received a report of a cluster of seven deaths in Maganjo Parish, Nabweru Subcounty, Wakiso District, Central Uganda. The case-patients presented with sudden onset of headache, vomiting, loss of eyesight, and loss of consciousness before death. All cases reportedly occurred after drinking alcohol. We conducted an epidemiological investigation to determine the cause of sudden deaths, identify risk factors for the deaths, and recommend control measures for the outbreak.

## 2. Methods

### 2.1. Setting

Wakiso District is located in the Central Region of Uganda and partly encircles Kampala, Uganda's capital city. The affected subcounties, Nabweru and Nangabo, are comprised of urban slums with limited access to public healthcare services. The main economic activity in the affected subcounties involves small-scale businesses.

### 2.2. Case Definition

We defined a probable case as onset of acute loss of eyesight and any of the following symptoms: vomiting, headache, nausea, dizziness, and loss of consciousness in a resident of either Nabweru or Nangabo Subcounty, Wakiso District, from 1 to 30 June 2017.

### 2.3. Case Finding

We reviewed the outpatient and inpatient medical records for the month of June 2018 at two private health facilities where the case-patients had sought care. With the help of community leaders and members of village health teams, we conducted active case finding in the affected subcounties. We interviewed survivors and next of kin of decedents at their homes. We held focus group discussions with local leaders in the affected villages on whether or not any similar patients or deaths had occurred in their communities.

### 2.4. Descriptive Epidemiology

We described the distributions of patients' clinical presentations and the onset of symptoms. We used the attack rate (AR) to evaluate the distribution of cases by age, sex, and village of residence. In calculating the ARs, we used the population projection data for 2017 based on the 2014 population census, provided by the Uganda Bureau of Statistics [4].

### 2.5. Hypothesis Generation

Using a structured questionnaire, we interviewed survivors and next of kin of decedents on case-patients' alcohol exposures two days prior to their onset of symptoms. We hypothesized that drinking “waragi” (especially from bars that were supplied by wholesaler *X*) was associated with this outbreak.

### 2.6. Case-Control Study

We conducted a case-control study to test the hypotheses generated during descriptive epidemiologic analysis and hypothesis-generation interviews. Because all case-patients were adults (>18 years) who had drunk alcohol, we selected controls among persons who drank alcohol and lived in the same neighborhood as case-patients. Controls were matched to case-patients by age, sex, and village. All case-patients (including the deceased) were included in the case-control study. Using a structured questionnaire, we interviewed both the case-patients and controls on the type of alcohol consumed, the place where they drank, and whether they had drunk alcohol during 8–20 June 2017. For the deceased case-patients, we interviewed their next of kin (e.g., spouses and siblings).

### 2.7. Laboratory Analysis and Postmortem

With the assistance of the Uganda Police Authorities, we collected samples of a locally distilled alcohol called “waragi” from the implicated bars and from wholesaler *X*, who supplied the implicated bars. We submitted the samples to the Government Analytical Laboratory for toxicological analysis. The “waragi” samples were first tested using the colorimetric test method to determine whether there were compounds other than alcohol. The presence of methanol was determined using the UV-vis spectrophotometric method [5].

### 2.8. Trace-Back Investigation

We conducted a trace-back investigation to identify the source of the implicated alcohol by interviewing the owners of the implicated bars. We conducted key informant interviews of “waragi” sellers and leaders of affected villages to identify at what point, how, and why the alcohol might have been contaminated.

### 2.9. Ethics Approval

This investigation was in response to a public health emergency and was therefore determined to be nonresearch. The MoH gave the directive and approval to investigate this outbreak. The Office of the Associate Director for Science, Centre for Global Health, CDC/Atlanta, also determined that this activity was not human subject research, and its primary intent was public health practice or a disease control activity (specifically, epidemic or endemic disease control activity).

## 3. Results

### 3.1. Descriptive Epidemiology

We identified 15 probable cases, with 12 deaths (case-fatality rate = 80%). The median age of the patients was 43 (range: 23–66) years. Patients presented with loss of eyesight, profuse sweating, vomiting, loss of consciousness, and other symptoms (Table 1).

**Figure 1:**
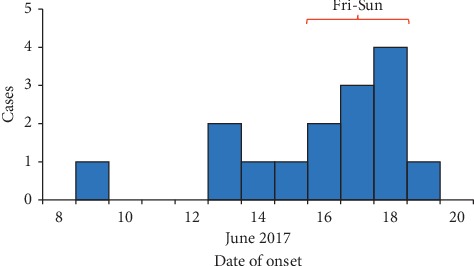
Methanol poisoning cases by onset date during an outbreak in Wakiso District, Uganda, June 2017.

The first case-patient developed symptoms on 9 June and the last on 19 June. The epidemic curve suggested a continuous common-source exposure lasting 11 days (Figure 1).

**Figure 2:**
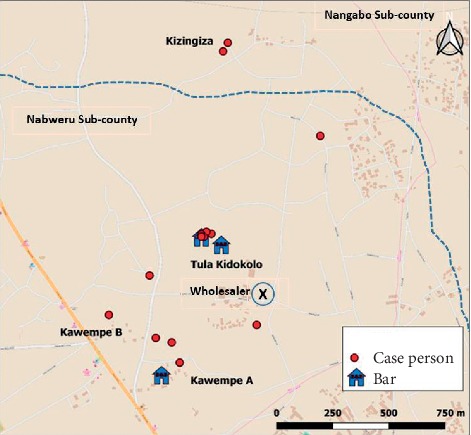
Geographic distribution of methanol poisoning cases during an outbreak in Wakiso District, Uganda, June 2017.

Men (AR = 7.6/10,000) were 3.6 times as likely to be affected as women (AR = 2.1/10,000). The outbreak affected the 36- to 55-year age group (AR = 17/10,000) more than other age groups. Cases occurred in the Nabweru (4 villages) and Nangabo (1 village) subcounties. Of the 15 probable case-patients, 12 (80%) resided around 3 bars in the area (Figure 2).

All case-patients had drunk alcohol before the onset of symptoms. The mean time between the most recent alcohol ingestion and development of symptoms was 12 (range: 3–24) hours. The mean time between the onset of symptoms and death was 19 (range: 1–23) hours.

Of the 15 case-patients, 9 (60%) sought medical care. The mean time between symptom onset and treatment was 4.1 (range: 1–9) hours for the case-patients who sought treatment. The mean time from symptom onset to treatment for the 6 deceased patients was more than twice as long as the 3 survivors (5.0 vs. 2.3 hours, *p*=0.18). Of the 15 patients, 11 (73%) did not receive intravenous ethanol, which should be the standard of care (including 5 who did not seek medical care at all), and all (100%) died; in contrast, 4 (27%) received ethanol treatment and 1 (25%) died (RR = 4.0, 95% CI: 0.73–22). Among the 4 patients who received ethanol treatment, the mean time from symptom onset to receiving the treatment was 3.5 (range: 2–7) hours.

### 3.2. Findings of Hypothesis-Generation Interviews

All 15 case-patients had drunk “waragi”; 87% of the case-patients drank “waragi” from at least 1 of the 3 bars supplied by wholesaler *X*. We therefore maintained our hypothesis that drinking “waragi” from bars that were supplied by wholesaler *X* was associated with this outbreak.

### 3.3. Case-Control Study Findings

87% of case-patients had last drunk “waragi” from a bar that was supplied by wholesaler *X* before the onset of symptoms, compared to 25% of controls (OR_M-H_ = 15; 95% CI: 2.3–106) (Table 2). Drinking alcohol packed in sachets and beer was not significantly associated with illness.

### 3.4. Laboratory Findings

All 10 “waragi” samples collected from the implicated bars and wholesaler *X* contained high concentrations of methanol (mean: 1200 mg/L; range: 77–2711 mg/L; compared with the safe level of <50 mg/L). Two of the samples contained 45 mg/L and 34 mg/L of esters, respectively, which were above the recommended safe level of 30 mg/L. Two other samples contained 87 mg/L and 57 mg/L of volatile acidity, which were also above the recommended safe level of 50 mg/L.

### 3.5. Trace-Back Investigation

The implicated alcohol was from Kasubi Market in Rubaga Division, Kampala District. This market served as a collection-distribution centre for goods from various parts of Uganda. A special area in the market was designated for selling “waragi” to distributors. Before distribution, routine alcohol testing was reportedly conducted using an alcoholmeter for ethanol concentration to assess its suitability and safety for human consumption.

We found that the implicated lot of “waragi” was from Kagadi District in western Uganda. The same lot was also distributed to various parts of Kampala and Wakiso districts, yet only Nabweru and Nangabo subcounties in Wakiso District were affected. Further investigation revealed that the implicated bars only obtained “waragi” from wholesaler *X*. Wholesaler *X* had only supplied “waragi” to the 3 bars in the area. When being interviewed, wholesaler *X* denied having added methanol to drinking alcohol. However, interviews of area alcohol distributors revealed that it was a fairly common practice for alcohol sellers to add methanol to drinking alcohol to improve its taste and to increase the profit margin. Most of the time, the amount of methanol added to alcohol was carefully controlled.

Police investigation found that wholesaler *X* did not have a license to sell “waragi”. Therefore, wholesaler *X* was arrested and prosecuted.

## 4. Discussion

This cluster of cases with a high case-fatality rate was caused by drinking a locally distilled alcohol adulterated with methanol. High levels of methanol were found in the implicated alcohol. Cases only occurred among patrons of 3 local bars, which were all supplied by a single wholesaler. Circumstantial evidence suggested that he likely had adulterated the alcohol before selling it to the bars, despite that the wholesaler denied this allegation.

Once ingested, methanol metabolizes into the highly toxic formic acid. Accumulated formic acid in the body results in metabolic acidosis, which inhibits cytochrome oxidase in the mitochondria, leading to histotoxic hypoxia [6]. The brain and the visual pathway are highly sensitive to formic acid, but other organs may also be seriously damaged, depending on the severity of metabolic acidosis. Our laboratory investigations also identified other compounds, including esters and volatile acids. However, these are usually found in distilled spirits and not known to cause death unless their concentrations are very high [7].

Methanol can be found in both alcoholic and nonalcoholic drinks as a natural product of fermentation [7]. Usually, the concentration of naturally occurring methanol in these drinks is well below the harmful level. Problems can arise when higher concentrations are formed during incorrectly managed distillation processes, but more particularly when methanol is deliberately added to informally produced spirits and illicit alcoholic drinks [3]. Industrial methanol is cheap (approximately $3/litre, compared with $5/litre for “waragi”) and widely available in Uganda [8]; moreover, adding methanol reportedly can make the “waragi” taste better, thereby boosting both the sale volume and the price and increasing the profit margin.

Suspected methanol poisoning outbreaks with fatal outcomes have been reported in Uganda [9]. However, no thorough investigations were conducted during those outbreaks. Outbreaks of methanol poisoning have also been reported in other parts of the world. In 2013, more than 1,000 patients were poisoned in Libya's capital city Tripoli, with a reported case-fatality rate of 10%. In Kenya, 2 methanol poisoning outbreaks affected approximately 341 and 126 persons, with case-fatality rates of 29% (100/341) and 21% (26/126), respectively [10]. Several methods currently exist to test the presence of methanol in alcoholic beverages. The validity of some of those methods is questionable [11]. Methanol testing strips, especially liquor methanol content rapid strips, are the simplest, fastest, and valid screening test. The advantages of the testing strips include instant results, ease of use, minimal training required, and cost-effectiveness [12]. They detect methanol content in drinks even when the quantity is small and can be used at any time by shop owners, hotels, regulatory personnel, and even spirit drinkers. However, this method is currently not yet available in Uganda.

To prevent death and severe sequelae due to methanol poisoning, ethanol should be administered intravenously as soon as possible after methanol ingestion [2, 13, 14]. Ethanol is an effective antidote to methanol poisoning and can prevent formic acid formation by competitively blocking the ADH. Ethanol has a higher affinity for ADH than methanol. Its serum concentration completely blocks the metabolism of methanol to formaldehyde [15]. Although another antidote, fomepizole, is equally effective [2], ethanol's wide availability in the community makes it a superior antidote for a prehospital “first aid” in the event of suspicious toxic alcohol ingestion. During outbreaks, patients often first seek medical care at small clinics. Therefore, awareness of clinicians at these small clinics on the importance of timely intravenous administration of ethanol can save precious time, leading to improved treatment outcomes [16].

The case-fatality rate during this outbreak (80%) was substantially higher than that during other outbreaks (around 30%) [10]. This high case-fatality rate likely was due to the lack of and delayed medical care. Among the victims that sought treatment, only 4 received intravenous ethanol treatment. Delayed medical care has been shown to contribute to high rates of sequelae and death during previous methanol poisoning outbreaks as well [17].

In Uganda, laws governing alcohol distillation and production have been established. The Uganda “Enguli” (Local Alcohol Manufacture and Licensing) Act, established in 1966, prohibits the manufacture, sale, and purchase of locally distilled alcohol without a license and states that any person who contravenes or fails to comply with any of the provisions of the Act commits an offence and is liable to conviction [18]. However, these laws have been inadequately enforced.

### 4.1. Limitations

We were unable to confirm the presence of methanol in the blood of the patients because the specimens had been spoiled when they arrived at the laboratory. However, the patients' clinical presentations, exposure history, and epidemiologic data made a strong case that this outbreak was due to methanol poisoning.

## 5. Conclusions

We concluded that this outbreak was caused by drinking the locally distilled alcohol, “waragi,” adulterated with methanol. We recommended enforcement of existing laws governing alcohol distillation and production, public health campaigns to promote awareness about methanol poisoning, introduction and use of methanol test strips by sellers to ensure product safety, and timely intravenous administration of ethanol to victims of methanol poisoning. We also recommended that the Uganda National Bureau of Standards should consider conducting random, impromptu quality-control checks of locally distilled alcohol and using the results to guide interventions, so as to deter alcohol producers and distributors from attempting to adulterate drinking alcohol. The relevant authorities incorporated the recommendations from this investigation and it is on this basis that from 2017 to date, no such occurrences have been reported.

## Figures and Tables

**Table 1:** Distribution of clinical features of 15 cases of methanol poisoning during an outbreak in Wakiso District, Uganda, June 2017.

Clinical feature	*n*	%
Loss of vision	15	100
Profuse sweating	11	73
Vomiting	10	66
Loss of consciousness	4	29
Headache	4	29
Nausea	2	14
Dizziness	2	14
Diarrhea	2	14
Fever	1	6

**Table 2:** Association between most recent exposure to alcohol and methanol poisoning among 15 case-patients and 60 control subjects during an outbreak in Wakiso District, Uganda, June 2017.

Alcoholic beverage last consumed during 8–20 June 2017		% cases (*n* = 15)	% control (*n* = 60)	OR_M-H_ (95% CI)
“Waragi”^†^ from a bar supplied by wholesaler *X*		87	25	15 (2.3–106)
Alcohol packed in sachets		73	53	2.3 (0.64–7.8)
Beer		60	73	0.45 (0.13–1.7)

^†^A locally distilled alcohol.

## Data Availability

Primary data were used to support the findings in this investigation. The dataset used in this investigation is available from the corresponding author upon request.

## Abbreviations

ADH: Alcohol dehydrogenase enzyme
AR: Attack rate
CDC: Centre for Disease Control and Prevention
CI: Confidence interval
HIV/AIDS: Human immunodeficiency virus/acquired immunodeficiency syndrome
MoH: Ministry of Health
ORM-H: Mantel–Haenszel odds ratio
UV-vis: Ultraviolet-visible.
